# Triggering Innate Immune Receptors as New Therapies in Alzheimer’s Disease and Multiple Sclerosis

**DOI:** 10.3390/cells10082164

**Published:** 2021-08-22

**Authors:** Pierre-Alexandre Piec, Vincent Pons, Serge Rivest

**Affiliations:** Neuroscience Laboratory, CHU de Québec Research Center, Department of Molecular Medicine, Faculty of Medicine, Laval University, 2705 Laurier Boul., Québec, QC G1V 4G2, Canada; pierre-alexandre.piec@crchudequebec.ulaval.ca (P.-A.P.); vincent.pons@crchudequebec.ulaval.ca (V.P.)

**Keywords:** monocytes, microglia, amyloid, innate immunity, Toll-like receptor, NOD2, muramyl-dipeptide, mCSF, MPL

## Abstract

Multiple sclerosis and Alzheimer’s disease are two complex neurodegenerative diseases involving the immune system. So far, available treatments provide at best mild improvements to patients’ conditions. For decades now, a new set of molecules have been used to modulate and regulate the innate immunity in these pathologies. Most studies have been carried out in rodents and some of them have reported tremendous beneficial effects on the disease course. The modulation of innate immune cells is of great interest since it provides new hope for patients. In this review, we will briefly overview the therapeutic potential of some molecules and receptors in multiple sclerosis and Alzheimer’s disease and how they could be used to exploit new therapeutic avenues.

## 1. Introduction

Alzheimer’s disease (AD) and multiple sclerosis (MS) are two major neurodegenerative diseases driven by an inappropriate response from innate and adaptative immunity [1]; although immunity is differently involved in both pathological processes [2]. This brief review focuses on the therapeutic potential of mCSF, MPL and MDP and their ability to modulate innate immunity. 

Microglia and monocytes are related to innate immunity; both arise from a common progenitor cell during embryogenic development [3]. Microglia invade the embryonic brain and then maturate, expand and colonize the brain [4]. Microglial cells in the brain take part in neurogenesis, synaptogenesis and synaptic pruning, brain development, brain homeostasis and brain defense [5]. Microglia continuously survey the brain with their motile processes and can quickly respond to an insult or infection [6], meaning that microglial cells are highly plastic; they can produce a large variety of molecules from inflammatory molecules such as tumor necrosis factor-α, reactive oxygen species and nitric oxide as well as trophic factors providing support to the brain, namely BDNF, IGF-1, arginase-1 and TGF-β [7].

On the other hand, monocytes arise and are produced in bone marrow (BM) throughout life [8]; unlike microglia, monocytes have a short lifespan [8]. In humans and mice, monocytes can be divided into three subpopulations, namely inflammatory/classical, intermediate and patrolling/non-classical distinguished by the expression of CD14, CD16 in humans and Ly6C in mice [8,9]. According to this classification, in humans (1) inflammatory monocytes express CD14^++^CD16^−^, (2) intermediate CD14^+^CD16^+^ and (3) non-classical CD14^−^CD16^++^, whereas in mice, (1) Ly6^hi^CCR2^+^ for classical, (2) Ly6C^int^CX3CR1^+^ intermediate and (3) Ly6C^low^CX3CR1^+^CCR2^−^. These populations are of interest since they display similar properties between the two species [10,11]. Indeed, classical monocytes respond rapidly to infection or injury. The recruitment of this population is CCR2 dependent. Classical monocytes can penetrate into the brain where they maturate into microglia-like cells and participate in the pathological process [12,13] since they are able to produce a wide range of inflammatory molecules such as TNF-α, Interleukin (IL)-1β, ROS, NOS, CCL2 and NLRP3 [14]. 

The other subpopulation of interest is patrolling monocytes, and the latter have unique patrolling behaviors depending on the expression of LFA-1 [15]; this integrin allows them to roll on the walls of blood vessels. Patrolling monocyte survival is highly dependent on the expression of Nur77, CD115 and CEBP. Moreover, they upregulate the transcription of genes controlling the cytoskeleton as well as CX3CR1 and CD115 [16]. Noteworthy, patrolling monocytes are not able to produce ROS suggesting that these immune cells do not participate in inflammation, and they seem to regulate the latter [17,18].

At this time, we will not discuss the role of intermediate monocytes since they are not as well characterized as the previously mentioned subpopulations, but they remain of great interest according to recent studies [19,20].

In recent decades, a growing number of studies have demonstrated that directing the response of monocytes and/or microglia is beneficial in the context of AD and MS [21,22]. To further understand why the modulation of innate immunity could be an interesting therapeutic avenue, in the next section we provide a brief overview of both pathologies and the implication of monocytes and microglia in the pathological process of AD and MS. 

## 2. Alzheimer’s Disease

AD is the most common form of dementia worldwide; the prevalence is constantly growing due to aging of the population and a lack of treatment able to slow the progression of the disease [23]. AD is partly characterized by the accumulation of amyloid beta (Aβ) in the brain, hyperphosphorylated tau protein, specific neuronal loss and activation of immune cells [24]. The most common symptom is short-term memory loss; as the disease progresses other symptoms such as personality changes, apathy and language problems emerge [25,26]. AD is a multifactorial disease several hypotheses have been developed to explain AD pathogenesis and the most studied and challenged is the amyloid hypothesis [27] which postulates that the abnormal accumulation of Aβ is the principal cause of neurodegeneration in AD [28,29]. The second hypothesis is the Tau hypothesis; Tau is a protein involved in the structure of microtubules and can be found in axons [30]. In AD tau is hyperphosphorylated and forms tangles that impair axonal transport [31]. The last well-known hypothesis is the cholinergic hypothesis, which postulates that cholinergic system degradation is the cause of AD [32]. However, none of these hypotheses can fully explain the disease as AD is not restricted to amyloid, tau or cholinergic neurons; AD can be considered a sex dependent disease since two thirds of patients are women, and this higher prevalence could be explained by genetics or hormones [33]. Indeed, one of the main hormones likely involved in AD is estrogen E2, which is found in the hippocampus; this molecule is involved in sex-specific behavior, synaptic plasticity and could have a neuroprotective role, but a past study found that E2 receptors could have a direct role in microglial reactivity by activation of the NLRP3 pathway [34]. However, the disease is more complex; researchers have identified two main forms: (1) late onset AD (LOAD), which is by far the most common and complex form in which the disease is triggered by genetic, epigenetic and environmental factors [35] and usually appears after the age of 65 [36]. The first discovered risk factor is APOE4; this mutation accounts for 50% of LOAD cases [37]. In a non-exhaustive manner, it is interesting to cite some other risk factors associated with the immune system, such as HLA DRB5/1, TREM2 and CR1 [38].

The other AD form is (2) early onset AD (EOAD), which is rarer but more aggressive [39]. EOAD is triggered by the mutation of genes involved in amyloid protein precursor (APP) or enzymes responsible for the cleavage of APP, namely PSEN1 and 2 [25]. The outcomes of these mutations differ from each other regarding the appearance of symptoms, the severity and the prevalence. Importantly, most mutations lead to an early onset long before the age of 65 [25].

Microglia are a central player in AD and are deeply involved in AD pathogenesis. Microglia roles in the disease have been extensively reviewed in [5,40,41,42,43,44]. Briefly, microglia lose their homeostatic phenotype and become highly reactive toward amyloid, resulting in the production of inflammatory factors, oxidative molecules and, over time, the downregulation of phagocytic abilities [45,46]. Indeed, microglia under homeostatic conditions synthesize neurotrophic factors such as NGF, BDNF and IGF-1. However, recent studies have shown that NGF and BDNF expression are decreased in AD patients, which may contribute to cognitive decline [47,48]. An important role of microglia is the engulfment and the clearance of debris. Phagocytosis of amyloid by microglia is mediated by TREM2 [49]; this receptor is important for microglia in healthy conditions since it promotes microglia survival, proliferation and reactivity [50,51,52]. In AD, a loss of TREM2 leads to an increase in plaque and a decreased number of amyloid positive microglia. Moreover, TREM2 has been identified as a strong risk factor since mutations such as R47H and R62H alter the interaction between TREM2 and amyloid [49], whereas the up-regulation of TREM2 accelerates the clearance of amyloid and promotes the production of neurotrophic factor from microglia [53]. However, TREM2 is not the only receptor that can bind amyloid. TLR4, a well-known pattern recognition receptor (PRR), is also deeply involved in amyloid phagocytosis and microglia reactivity since amyloid is recognized as a damage-associated molecular pattern by TLR4, resulting in an overproduction of inflammatory cytokines and chemoattractant molecules such as CCL2; hence the latter attracts inflammatory monocytes and facilitates their infiltration into the brain, where they transform into microglia-like cells and participate in amyloid removal [54]. Microglia-like cells are more effective at clearing amyloid, but on the other hand they exacerbate neuroinflammation [55,56]. Additionally, TLR4 enhances the phagocytosis on a p38, CD36 dependent manner and may have a neuroprotective role [57]. These findings are corroborated by genomic studies linking TLR4 polymorphism to AD, suggesting that a defect in microglia reactivity could be one of the causes of AD [58]. Importantly, continuous exposure of microglia to amyloid leads to an exacerbated production of inflammatory factors and neurotoxic molecules [59] and the downregulation of phagocytosis markers [60]. It is worth mentioning that it is not only monocytes and microglia that participate in neuroinflammation, as neutrophils can infiltrate the brain and participate in inflammation and trap amyloid using their neutrophil extracellular traps. In parallel, astrocytes are important in the AD pathological process, and a recent study has shown that astrocyte gene expression is context-dependent and can vary between acute CNS injury versus neurodegenerative disease in mice [61]. In neurodegenerative contexts, there is an increase in genes coding for extracellular matrix proteins in astrocytes along with a decrease in *S1pr1* and *Sod2* genes that are an immune modulator and antioxidant factors, respectively [61]. A recent report raised the question regarding reactive astrocytes and their possible involvement in the pathogenesis process, since it is clear that the modifications of signaling pathways participate in disease progression [62].

There are currently several drugs on the market targeting cholinesterase, an enzyme involved in the degradation of acetylcholine, and glutamate receptor antagonists aiming to decrease the excitotoxic effects of glutamate, but their effects on the disease remain marginal [63]. As previously mentioned, amyloid participates in the pathology course and clinical trials addressing the amyloid hypothesis account for 22.3% of studies, followed by the cholinergic hypothesis (19%) that highlights the loss of cholinergic neurons as the starting point of AD [32]. Many treatments in trials focused on amyloid involved monoclonal antibodies targeting proteolytic enzymes responsible for APP cleavage or amyloid deposits [64,65]. The vast majority of these therapies failed in phase III in a non-exhaustive manner (e.g., solanezumab (Eli Lilly, Indianapolis, IN, USA), Crenezumab (Roche/Genetech/Ac Immune, Basel, Switzerland), Gantenerumab (Roche, Basel, Switzerland) [32]. These antibodies succeeded in reducing amyloid load but failed to improve cognitive decline. Moreover, they caused cerebral micro-hemorrhage and blood–brain barrier disruption and exacerbated neuroinflammation. Of interest, Aducanumab (Biogen, Cambridge, MA, USA), which has a high affinity for neurotoxic oligomeric species, has been partly authorized by the FDA, although its efficacy remains disputable [66]. Recently, the HAE-4 antibody targeting APOE4 did not display the aforementioned side effects in mice and seems effective at reducing amyloid in parenchyma and blood vessels [67].

These clinical trials were not made in vain, since they have shown that it is possible to reduce the amyloid load using innate immunity, but amyloid burden is not the only factor to focus on since reducing amyloid load does not improve cognition [63,68]. Interestingly, in rodents such as APP_swe/PS1_ mice a reduction in amyloid burden is associated with a better ability to execute cognitive tasks, which is interesting but raises questions about the relevance of our models and the inclusion criteria of clinical trials [69,70,71,72,73]. Indeed, rodents do not develop AD and mouse models of AD are less complex and mimic the EOAD. Moreover, genetic modifications leading to the accumulation of amyloid and behavioral deficits are in poor accordance with the real-world pathology [74,75]. It is noteworthy that a lack of efficacy of antibody therapy was also reported in dogs [76], raising questions about the adequacy of our rodent models. It is necessary to reduce the oligomeric load in the brain to decrease the neuroinflammation, but it is also necessary to enhance the support of tissues and maintain the blood–brain barrier integrity [77,78]. In this short review, we propose another mechanism of action since reducing amyloid load in humans does not provide beneficial outcomes regarding cognitive decline. We believe that the next generation of treatment should focus on direct modulation of immune cells by changing their state of activation and finely modulating the production of molecules from these cells as we will discuss further.

## 3. Multiple Sclerosis

MS is an chronic autoimmune, inflammatory, neurodegenerative disease [79]. The pathology usually appears between 20 and 40-year-old and impairs the sensation, motor and cognitive functions [80,81]. MS is also sex dependent, since it affects women more than men at a three-to-one ratio; the disease has different forms and the most common is the relapsing-remitting (RR), which involves alternation between periods of crisis and remission [82]. A crisis period refers to acute demyelination (DM) partly mediated by the immune system attacking the myelin and nerve fibers, whereas the remission period refers to spontaneous remyelination (RM). However, RM is incomplete or fails over time [83]. MS is clinically characterized by three different stages: (1) a preclinical stage, conditioned by genetic and environmental factors; (2) the clinical stage, which is an alternation between DM/RM periods; (3) the progression, during which the condition of the patient worsens. The latter is characterized by progressive neurodegeneration with a slight inflammatory component [80]. Importantly, 80% of the RR form evolves in the secondary chronic-progressive form where relapses are not necessarily apparent [84]. MS pathological features include blood–brain barrier breakdown, inflammation associated with reactive glial cells such as microglia and astrocytes and activation of lymphocytes and DM together with oligodendrocyte and neuronal loss [85]. The disease also has a genetic component since the first allele identified as a risk factor was HLA-DRB. Over the last decade, wide genome association studies using single nucleotide polymorphism compared MS patients with healthy persons; these studies helped to identify over 50 loci associated with the immune system that might be involved in disease onset and progression [86,87]. Interleukin (IL) 2 and 7 receptors, CXCR5, IL-12A, IL-12β and co-stimulatory molecules CD80, 86 and 37 are among these genes [88].

The environment is also believed to have a great impact on MS onset. In 2011, Simpson and colleagues published a study showing that the prevalence of MS is associated with geographic latitude [89]. Indeed, high latitudes are generally less exposed to sunlight, which is essential for the synthesis of vitamin D3, and a low level of vitamin D3 has been identified as a risk factor for MS onset [90]. Additionally, epidemiologic studies have demonstrated that high exposure to sunlight is inversely correlated with the risk of developing MS [88,91]. The vitamin D receptor is present in almost every immune cell and the active form of vitamin D3 plays an essential role in lymphocyte regulation, reactivity and proliferation [92,93]. Some data have linked a deficiency in vitamin D to a defective immune system function and a predisposition to autoimmune diseases [94,95]. However, the effect of vitamin D3 on MS is still debated and remains to be fully demonstrated [96].

Several animal models have been established to study the physiopathology of MS. The experimental autoimmune encephalomyelitis (EAE) is the most studied model in which autoimmunity to myelin components is induced [97]. (1) The first description was made on monkeys using rabbit brain extracts to prime immune cells. Later, Freund’s adjuvant and pertussis toxin were used to prime the immune system and to induce blood–brain barrier breakdown. Myelin oligodendrocyte glycoprotein or myelin basic protein or proteolipid protein were then used as immunogen molecules. EAE is characterized by an ascending paralysis [98]. (2) The cuprizone model is a model of DM/RM. Cuprizone is a copper chelator mostly used to study the role of the innate immune system in DM/RM and the fate of oligodendrocyte precursor cells (OPC) since cuprizone causes specific oligodendrocyte cell death [99]. Mature oligodendrocytes are the target of cuprizone, and intoxicated animals display drastic oligodendrocyte loss [98]. (3) Lysolecithin is another toxic agent that activates phospholipase A2 and triggers focal demyelination when injected into the spinal cord. The molecule is interesting since it induces a rapid DM without extensive damage on cells and axons; importantly, the demyelination is not mediated by the immune system [98]. Finally, (4) the delayed-type hypersensitivity (DTH) model consists of cell-mediated immune reactions driven by T cells. This model is not mediated by antibodies, as the reaction is caused by the interaction between innate and adaptative immunity; resulting in the release of inflammatory mediators from reactive T cells [100,101].

Monocytes and microglia are key players in MS and their modulation is a promising therapeutic avenue. Microglia are a double edge sword in MS since they participate in DM and RM via a large panel of molecules and their ability to uptake myelin [82]. Briefly, during DM microglia produce inflammatory factors and oxidative molecules such as ROS and NO, which are the major sources of damage for neurons and oligodendrocytes (and their precursors) [102]. Microglia and macrophages are the most abundant immune cells within MS lesions; however, microglia dominate the lesion at an early stage and during expansion of the lesion whereas macrophages are more present after the initial damage is done [103,104]. The role of microglia in the initiation of lesions was debated and some studies revealed that reactive microglia are found before the onset of EAE. Additionally, the EAE model revealed that the ablation of microglia attenuate oxidative stress and the severity of DM [103,105]. Indeed, reactive microglia express CD14 and MHC, which participate in the activation of T cells and produce MCP-1, CCL5 and IL-8 in a non-exhaustive manner, which contribute to the recruitment of T cells, macrophages, neutrophils, monocytes and dendritic cells, which increase inflammation and damage [85]. Innate immunity stimulates the adaptative immunity by presenting antigen and the release of cytokines [16]. Two different populations of lymphocytes are mobilized, namely CD8 T cells, which is the dominant lymphocyte subset in MS, and CD4 T cells, which represent 20–30% of total T lymphocytes [106,107]. According to the stimuli, the response from T cell differs. There are three major states of activation. (1) The main pathogenic subset is Th1, which secretes interferon-γ to activate natural killer cells and macrophages and then iNOS and MHC expression. (2) The Th2 subset promotes an anti-inflammatory response, whereas (3) Th17 favors the recruitment of neutrophils and promotes the activation of innate immunity [82]. 

RM is spontaneous and occurs simultaneously with DM or following DM [108]. A major actor in RM are microglia, which change their panel of cytokines and synthesize neurotrophic, anti-inflammatory and growth factors aiming to stimulate oligodendrogenesis, neurogenesis and therefore RM [82]. However, the newly formed myelin differs from the original [83]. Studies have also found that CX3CR1 knock-out mice have a greater extent of myelin debris and impaired RM due to the ineffective function of microglia [109]. Interestingly, in MS microglia drives the differentiation of OPCs and the proliferation of oligodendrocytes by phagocytosing myelin and through the production of IGF-1, FGF-2 and IL-4, which are important for oligodendrocyte proliferation [81,110]. All these data suggest that modulating immunity at different time points of the disease could ameliorate symptoms or delay their onset.

Several clinical trials have been carried out to assess the modulation of immunity in MS using newly synthesized molecules or antibodies. Some of them are promising, and in a non-exhaustive manner we can cite ocrelizumab, a human monoclonal antibody targeting CD20 on the surface of mature B cells, approved in 2017 [111]. The antibody is found to be efficient against relapses and can silence the progression of relapsing MS. The effect of ocrelizumab relies on the reduction of antigen presentation from B cells to T cells, and it also modulates the secretion of pro-inflammatory molecules from B cells and their reactivity. Along the same lines, rituximab, another anti-CD20, displays similar effects on the relapsing form of MS [23].

Fingolimod is a molecule approved as therapy for the relapsing form. The latter is an inhibitor of sphingosine-1-phosphate receptors, preventing the infiltration of adaptative immune cells into the CNS [112]. The drug has shown important effects in reducing the relapses during a phase III study [113]. However, the treatment presents some adverse events such as bradycardia and lymphopenia [112]. Glatiramer acetate and IFN-β have a mild impact on relapses. Glatiramer acetate alters the balance between pro-inflammatory and regulatory cytokines, whereas IFN-β downregulates inflammation and major histocompatibility complexes and inhibits T cell proliferation and prevents their infiltration into the brain [23]. Briefly, other molecules are on the market such as dimethyl fumarate, a disease modifying treatment that is recommended for active relapsing MS. The molecule affects T cell populations and enhances Th2 response. However, its role within the CNS of patients remains to be elucidated [114]. Natalizumab is used in RR form, the monoclonal antibody prevents the infiltration of leukocytes into the CNS by acting as an antagonist of integrin. The treatment based on natalizumab is broadly used [115]. Finally, alemtuzumab, is a monoclonal antibody that binds to CD52, leading to a long-lasting depletion of CD52 positive B and T cells. It is indicated for RR form [116].

All these therapies participate in improving or delaying the disease. However, none of them can cure MS. Recently, some molecules that modulate the innate immune system have shown promising results in animal models of MS, namely mCSF and MDP.

## 4. New Therapeutic Avenues for Neurodegenerative Diseases

### 4.1. mCSF

mCSF and its receptor CSFR1 are broadly expressed by myeloid cells. The axis triggers activation, migration and development of monocytes and monocyte-derived cells [117]. CSFR1 has been extensively studied in the context of neurodegenerative diseases since its modulation is thought to be a potential therapeutic avenue [118]. Several studies have demonstrated the beneficial effects of such therapy in vivo and in vitro in AD and MS [54,118,119]. In 2018, Laflamme and colleagues used mCSF in a cuprizone model to assess the effect of the cytokine on DM/RM and they showed that the immune response was increased in corpus callosum following mCSF injections. The treatment also extended myelin coverage associated with an increased number of oligodendrocytes, suggesting a better clearance of myelin debris and a greater RM rate. Interestingly, authors have shown that mCSF injections trigger the expression of scavenger receptors and consequently ameliorate the phagocytic ability of microglia. Interestingly, aberrant myelin debris depositions associated with an impaired microglial response was observed in CSF1R-deleted mice [120], suggesting a strong protective role of mCSF/CSF1R axis in DM/RM processes.

Microglia reactivity and phagocytic response during DM is critical for proper RM, since myelin debris impedes the proliferation and maturation of oligodendrocyte precursors [109,121]. MCSF injections stimulate the phagocytose of myelin and drive a microglial response during acute demyelination. Interestingly, it has been shown in humans that the mCSF/CSF1R axis is downregulated within MS lesions, suggesting that stimulated cells using mCSF injections could provide better outcomes in humans [122]. Conversely, a CSF1R blockade was shown to be beneficial in an EAE model [123,124]. To evaluate the role of CSF1R in EAE, Uemura and colleagues used Ki20227, a selective inhibitor of CSF1R signaling pathways, and reported a delayed onset and a lower EAE score compared to the non-treated group using both prophylactic and therapeutic regimens. The suppression/reduction of clinical symptoms is associated with a significant decrease in T-cell proliferation and the suppression of IFN-γ and TNF-α production. The drug acts on myeloid cells by impeding their reactivity, but the mechanism underlying the beneficial effect of CSF1R inhibition is not fully understood yet. However, Ki20227 decreases the gene expression of molecules involved in the inflammatory cascade, including NLRP3, caspase-1 and NF-κB, while increasing IL-10 and arginase-1 mRNA expression [125]. These results suggest that CSF1R is deeply involved in the recruitment of adaptative immunity and inflammatory response [126].

Recently, Hagan and colleagues proposed that the CSF1R pathway could regulate MS, and to demonstrate their hypothesis they used a CSF1R inhibitor on primary murine microglia to study its impact on the proliferation and production of pro-inflammatory molecules. The results revealed that CSF1R inhibitors alter the proliferation of microglia and decrease the secretion of MCP-1 and IL-12p40, two major chemoattractant molecules. However, by inhibiting CSF1R they did not find a decrease in cell viability, which is quite interesting since most inhibitors of CSF1R lead to a massive depletion of microglia. Overall, results from Uemura et al. and Hagan et al. point out the role of CSF1R in inflammatory responses and in the recruitment of peripheral immune cells in MS [127]. Other molecules inhibiting CSF1R, namely PLX, are largely used in different pathologies, such as rheumatoid arthritis, glioma, and AD, but there remains a vivid debate about the real efficacy and effect on myeloid cells of such a molecule [21,128]. Indeed, a high dose of either PLX5622 or 3397 induces a reduction in OPC number in vitro and ex vivo, raising the question of a potential cross-effect of these molecules [128,129]. Interestingly, Wlodarczyk and colleagues found similar results regarding the improvement of EAE mouse conditions when they stimulated CSF1R. The study showed that mCSF injections lead to the expansion of a specific microglial subpopulation distinguished by the overexpression of CD11c. Importantly, this particular microglial subset is a major source of IGF1, a growth factor important for myelination and the recruitment of OPCs to the MS lesion [130]. Additionally, CD11c^+^ microglia number increases during neuroinflammation in both EAE and cuprizone animal models [131,132]. These microglia are believed to regulate inflammation since CD11c^+^ microglia are a poor inducer of Th1 and Th17 responses. Additionally, chemoattractant axes, namely MCP-1/CCR2, have pro-inflammatory effects in general. Interestingly, some researchers have proposed that CCL2 upregulation via CSF1R can promote neuroprotection in a CCR2-independent manner since the latter is not expressed by microglia [132,133].

mCSF is known to modulate microglial response as well as the infiltration of monocytes, which then differentiate into microglia-like cells [54,133]. These newly formed microglia-like cells more potently uptake amyloid [54]. The role of mCSF/CSF1R axis is paradoxical as it improves the condition in cuprizone models, leading to a better myelin clearance and a greater RM rate, whereas the inhibition of the axis via different molecules in EAE model delays or suppresses symptoms. These results are not antonymic since both MS models are different and require distinctive populations of immune cells. Indeed, the cuprizone model preferentially stimulates myeloid cells and mCSF-induced stimulation of innate immune cells seems to improve their phagocytic response, myelin clearance and RM [134,135]. In contrast, the cytokine may be harmful when the adaptative immunity is involved in the EAE model. 

The modulation of innate immunity via the mCSF/CSF1R axis is a potential therapeutic avenue in both MS and AD. However, the role of the axis requires deeper study regarding some conflicting results between studies and pre-clinical models.

### 4.2. MDP

Muramyl-dipeptide (MDP) is a component of bacterial cell walls that binds to a PRR, namely nucleotide-binding oligomerization domain 2 (NOD2) [136]. NOD2 is involved in different chronic inflammatory pathologies such as Crohn’s disease and Blau syndrome [137,138]. MDP is seen as a potential new therapeutic avenue since the molecule via NOD2 induces a switch in the population of monocytes from inflammatory to patrolling subset. The increased number of patrolling monocytes could be beneficial in MS since non-classical monocytes are able to modulate inflammation and contribute to tissue repair [16]. A recent study by Lessard and colleagues demonstrated that the switch induced by MDP is mediated by NOD2 and NurActually mice lacking NOD2 upon MDP injection do not display a switch. Moreover, Nur77 knockout mice have a defect in non-classical monocyte production, suggesting that NOD2-Nur77 are the principal effectors of MDP [139,140]. Nur77 is an orphan nuclear receptor involved in different cell functions such as apoptosis, organ development and metabolism [141]. AP-1 and CREB regulate the transcription of Nur77 [142]. The exact mechanism involved in the switch is not fully understood. Several studies have been conducted to assess the role of patrolling monocytes during inflammation, and it seems that the activation of Nur77 is able to modulate the inflammation and repair processes by overexpressing IL-4 and IL-10, suggesting a potential therapeutic role of such monocyte subsets in inflammatory diseases [17,143,144]. Indeed, inflammatory monocytes are increased within the first hours after immunization in an EAE model. However, it has been shown that a lack of Ly6C^hi^ prevents or reduces EAE progression suggesting that a switch of population during a crisis could be beneficial to prevent inflammation [145]. In our lab we have recently demonstrated that early injections of MDP have a strong and beneficial effect by delaying the prevalence of the disease. This outcome is driven by the upregulation of Ly6C^low^ monocytes and the drastic diminution of Ly6C^hi^ in the blood of EAE mouse models [146]. Moreover, MDP treatment after immunization was able to prevent EAE progression. Interestingly, we observed a decrease in the number of infiltrating cells, namely CD8^+^, CD4^+^, CD3^+^, neutrophils and inflammatory monocytes, suggesting that MDP could be used in prophylaxis or during a crisis. However, MDP-treated mice did not display difference regarding the demyelination rate or oligodendrocyte number in cuprizone models [146].

Despite the low number of available studies on MDP in MS we can hypothesize according to the literature that non-classical monocytes are beneficial in MS in various ways. (1) The major histocompatibility complex class II is less expressed by non-classical monocytes, indicating that this specific subset is less prone to stimulation by T cells [147]. (2) The modulation of inflammatory response since non-classical monocytes do not produce reactive oxygen species and promote tissue repair via Nur77 activation, which is important to control the inflammatory response since it inhibits the production of TNF-α, IL-1β and oxidized lipids [148,149]. (3) Ly6C^hi^ have been found to be one of the major contributors to MS lesions and MDP could lower the impact of MS by downregulating this population [150].

These results provided by MDP are encouraging and need to be confirmed by further studies.

Regulating the inflammatory response could also be a promising strategy in AD. Our group has recently demonstrated that repeated injections of MDP in a mouse model of AD from three months old to six months old once a week can prevent the appearance of symptoms [151]. We hypothesized that increasing the pool of patrolling monocytes could decrease the amyloid load in brain blood vessels by triggering a sink effect between the brain and the vasculature. The latter is well defined but remains controversial, with evidence either in favor or against this mechanism [65,152]. The study has shown a delayed cognitive decline associated with a stable increase in patrolling monocytes and a drastic diminution of inflammatory monocytes in the blood over time. Interestingly, the effect of repeated injections of MDP was still effective in six-month-old mice and we did not find health issues suggesting that the molecule is safe in mice even for three consecutive months [151]. These results also suggest that the cognitive improvement in the MDP group could be attributed to the sink effect since MDP-injected mice presented a higher expression level of LRP. This receptor is able to transport amyloid through the blood–brain barrier and, interestingly, LRP1 expression is downregulated in AD [153,154]. The cognitive improvement is also associated with a higher expression of the synaptic marker PSD-95 compared to APP mice at six months [151]. Altogether, these results demonstrated a strong beneficial role of MDP in AD, but further studies are needed to unveil the underlying mechanisms.

### 4.3. Modulation of TLR4 Response in AD

TLR4 is a key PRR for the activation of immune responses. TLR4, MD-2 and CD14 recognize PAMPs like LPS. Upon binding, LPS leads to the recruitment of intracellular TIR-domain-containing adaptors, including MyD88, which is followed by the activation of MAP kinases and the activation of NF-κB, triggering the transcription of innate immune genes, such inflammatory cytokines, chemokines, adhesion molecules and prostaglandins [155,156].

On the other hand, the non-pyrogenic LPS derivative MPL displays a low toxicity for cells. Importantly, MPL is a weak inducer of the inflammatory response [116]. MPL poorly triggers MyD88-dependent pathways, impeding TLR4/MD-2 dimerization, which diminishes TNF-α production [157]. In 2013, our group used MPL as a therapeutic tool against AD and we found that MPL has powerful and beneficial effects in an AD mouse model. Indeed, Michaud and colleagues injected either MPL or LPS into AD mice from three months old to six months old. As expected, we found a decreased inflammatory response from microglia with an enhanced phagocytic capacity in MPL-injected mice compared to the LPS group. This improvement of phagocytosis could be a consequence of the reactivation of the p-38/SRA pathway, which is impaired in both AD patients and APP mice [22,158]. These results demonstrated that MPL can effectively modulate the immune response and improve the pathological feature of AD [159,160]. 

In the same vein, Jian Zhou and colleagues examined a potential link between AD and systemic inflammation using a high dose of LPS in 7-month-old APP mice and in a BV2 cell line. They reported that LPS leads to microglial over-reactivity followed by an increased neuronal loss in the cortex associated with decreased phagocytic activity. Furthermore, a high dose of LPS worsened the cognitive decline of AD animals. The authors also found that the microglial capacity to engulf amyloid is inversely correlated with their activation state [161]. These findings showed that high exposure to LPS leads to an inappropriate microglial response, suggesting that a fine stimulation could be more beneficial [161].

Recently, new studies have demonstrated that the response to LPS is dependent on the dose and method of injection. Yousefi and colleagues reported that the pre-treatment of immune cells by a low dose of MPL or LPS seems to reprogram microglia toward a neuroprotective phenotype with an increase in anti-inflammatory molecule production such as INF-β, Arg1, Mrc1 and IRF3 [162,163,164]. Interestingly, INF-β can modulate the inflammatory response and leads to the activation of TREM2/DAP12 complexes that are crucial for amyloid uptake by microglia. On the other hand, Arg1 modulates the inflammation via the STAT6/Arg1 axis and can protect neurons by dampening ROS production and promoting extracellular matrix repair [130,165,166]. Additionally, Wendeln and colleagues have found that a low dose of LPS induced profound epigenetic reprogramming in microglial cells by enhancing the phagocytosis of amyloid, the production of neurotrophic factors and inhibition of IL-1β synthesis [167,168]. Recently, Pourdadie and colleagues used a low dose of MPL and LPS to prime microglia in an AD rat model and they found a modulation of anti-inflammatory molecules such as IL-10, arg1 and TGF-β in all groups of rats whereas solely LPS-injected rats displayed downregulation of TNF-α [169]. These results confirm that both TLR4 agonists induce different signaling pathways regarding the inflammatory response; however, inflammatory molecules seem to be necessary for proper microglial reactivity. Furthermore, they assessed the effect of a low dose of MPL and LPS on plaque numbers and found a lower number of plaques in MPL-treated mice, which was associated with cognitive improvement [22,169]. TLR4 ligands lead to an increase in phagocytosis by upregulating FPR2 in the cortex and hippocampus, which is a G protein-coupled receptor involved in amyloid chemoattraction and internalization [170,171]. Altogether, these studies support previous findings that microglia response is driven by MPL and that a low dose of LPS is beneficial in AD [118,170,171,172].

## 5. Conclusions

**Figure 1:**
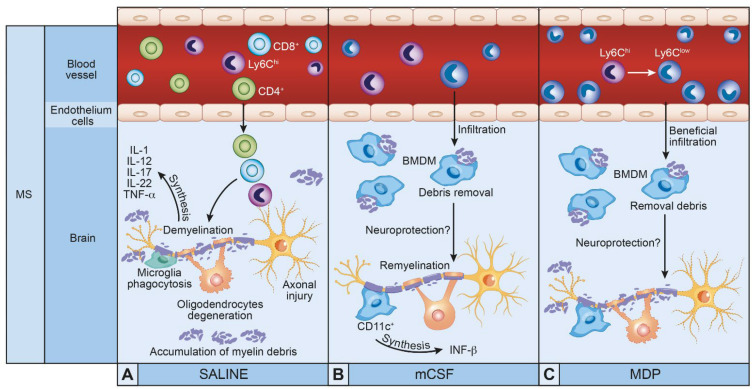
Therapeutic molecules modulating innate immune cells in multiple sclerosis (MS): (**A**) Cellular responses in the presence of myelin debris and hyperactivation of innate immune cells against myelin leading to oligodendrocyte degeneration. (**B**) Injections of macrophage colony stimulating factor (mCSF) trigger microglial proliferation and peripheral infiltration, improving myelin debris removal and remyelination and regeneration of oligodendrocytes. (**C**) Injections of muramyl dipeptide (MDP) induce the switch of monocytes Ly6C^hi^ to Ly6C^low^, improving myelin debris removal.

**Figure 2:**
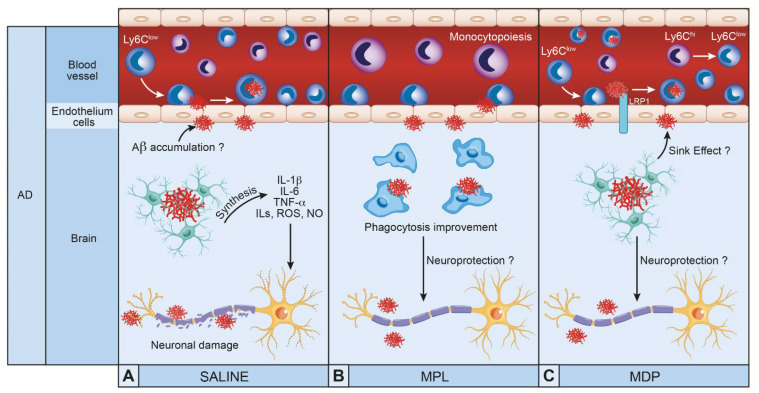
Therapeutic molecules modulating innate immune cells in Alzheimer’s disease (AD): (**A**) Accumulation of amyloid beta (Aβ) in the brain over-activates microglia and induces the synthesis of inflammatory cytokines (IL-1β, IL-6, TNF-α, ROS and NO) implicated in neuroinflammation and neuronal damage. (**B**) Injections of monophosphryl lipid A (MPL) polarize microglia into a neuroprotective aspect and improve microglia phagocytosis toward Aβ. (**C**) Injection of muramyl dipeptide (MDP) convert inflammatory monocytes (Ly6C^hi^) into an anti-inflammatory aspect (Ly6C^low^) with crawling phenotype. LRP1 transports amyloid through endothelial cells to the luminal side where patrolling monocytes can remove amyloid.

MS and AD are two major neurodegenerative diseases. Despite their complexity, several therapeutic molecules have been approved but unfortunately, they do not cure the disease. Researchers tested different molecules focusing on symptoms without strong improvements, nonetheless, another target has emerged in this decade. Immunomodulatory therapeutics promoting the elimination of Aβ by scavenger cells such as monocytes, macrophages, and microglia could be valuable tools in the fight against AD and MS. These new molecules have demonstrated their effects by modulating the immune response and therefore delaying the progression of pathologies (Figure 1 and Figure 2). The modulation of the innate immune system restores monocyte and microglial functions, and this strategy seems to be a new valuable therapeutic avenue to treat both MS an AD. It is important to note that these molecules do not cure the diseases; however, they are good candidates to treat and delay AD and MS. 

## Data Availability

Not applicable.
